# Serum Ceramide Species Are Associated with Liver Cirrhosis and Viral Genotype in Patients with Hepatitis C Infection

**DOI:** 10.3390/ijms23179806

**Published:** 2022-08-29

**Authors:** Marcus Höring, Georg Peschel, Jonathan Grimm, Sabrina Krautbauer, Martina Müller, Kilian Weigand, Gerhard Liebisch, Christa Buechler

**Affiliations:** 1 Institute of Clinical Chemistry and Laboratory Medicine, University Hospital Regensburg, 93053 Regensburg, Germany; 2 Department of Internal Medicine I, University Hospital Regensburg, 93053 Regensburg, Germany; 3 Department of Internal Medicine, Klinikum Fürstenfeldbruck, 82256 Fürstenfeldbruck, Germany; 4 Department of Gastroenterology, Gemeinschaftsklinikum Mittelrhein, 56073 Koblenz, Germany

**Keywords:** very-long-chain ceramides, long-chain ceramides, HCV genotype, direct-acting antivirals, liver cirrhosis, diabetes mellitus

## Abstract

Hepatitis C virus (HCV) infection affects ceramide metabolism, and, here, we have evaluated associations of eight serum ceramide species with viral load, viral genotype, and disease markers in 178 patients with chronic HCV. In this cohort, ceramide d18:1;O2/16:0 was higher in the serum of the 20 diabetic patients compared to the patients without this complication. Moreover, ceramide d18:1;O2/24:0 was negatively correlated with age. Of note, all but ceramide d18:1;O2/16:0 and 26:0 were diminished in the serum of patients with liver cirrhosis and, with the exception of ceramide d18:1;O2/16:0, were negatively correlated with the model for end-stage liver disease (MELD) score. Most of the serum ceramides are carried in low-density lipoprotein (LDL), which rises following effective direct-acting antiviral (DAA) therapy. Ceramide d18:1;O2/24:0 recovered in parallel with LDL, whereas ceramide d18:1;O2/18:0 declined. Genotype-3-infected patients had the lowest ceramide levels, which were comparable to other genotypes after DAA treatment. Notably, ceramide d18:1;O2/23:0 and 24:0 were negatively correlated with the MELD score in patients with liver cirrhosis at the end of DAA therapy. Long-chain (LC) ceramides show adverse effects, whereas very-long-chain (VL) species have protective functions in the liver. The ratio of VL/LC ceramides was higher in non-cirrhosis patients than cirrhosis patients and further increased at the end of therapy in this subgroup. In summary, our study shows that serum ceramide levels are related to liver cirrhosis and viral genotype. Whether the more favorable serum ceramide profile in non-cirrhosis patients, before and after DAA therapy, is of pathophysiological importance needs further investigation.

## 1. Introduction

Chronic hepatitis C virus (HCV) infection causes liver inflammation, which progresses to liver fibrosis and cirrhosis in most patients [1].

It has been established that HCV infection affects the patients’ lipid metabolism. Serum low-density lipoprotein (LDL) levels of HCV patients are reduced [2,3]. Among HCV infected patients, decreases in LDL were generally larger among genotype-3-infected patients, intermediate in genotype-1-infected patients, and lowest in genotype-2-infected patients [4]. Normalization of LDL shortly after successful anti-viral therapy indicates viral infection as the main cause of hypolipidemia [5,6,7]. This increase of LDL was particularly evident in genotype-1-infected and genotype-3-infected patients [8].

Reduced serum LDL is in part the consequence of impaired hepatic very-low-density lipoprotein (VLDL) release. Diminished secretion of lipids by the liver may cause hepatic lipid deposition and liver steatosis. Notably, fatty liver is a relatively common diagnosis in HCV patients and is most prevalent in genotype-3-infected patients [2]. In addition to viral effects on lipid metabolism, insulin resistance induced by HCV infection has a defined role in the development of liver steatosis [2,9]. Elimination of HCV does not, however, consistently improve liver steatosis, and insulin resistance due to concomitant metabolic disease may be the underlying cause of fatty liver disease in some patients [10,11].

LDL is composed of different lipid classes and contains cholesterol, triglycerides, phosphatidylcholine, phosphatidylethanolamine, lysophosphatidylcholine, and sphingolipids [12].

Ceramides are precursors of more complex sphingolipids and are relatively well-studied lipids [13,14,15]. LDL carries about 50% of lipoprotein sphingomyelin and 60% of lipoprotein ceramides [12]. Accordingly, serum levels of ceramide species are positively correlated with serum cholesterol and triglycerides [16]. Ceramide d18:1;O2/24:0 is the most abundant species in LDL and makes up nearly 50% of the LDL-carried ceramides. The next most common species are ceramide d18:1;O2/24:1, 23:0, and 22:0. Ceramide d18:1;O2/16:0 makes about 8% of LDL ceramides, while ceramide d18:1;O2/18:0 and 20:0 make less than 2.5% [12].

According to the low levels of LDL in chronic HCV, ceramide d18:1;O2/24:0 was reduced in comparison to healthy controls. Ceramide d18:1;O2/14:0, 16:0, 18:0, 20:0, and 24:1 did not differ between the controls and HCV-infected patients [17].

Notably, ceramide d18:1;O2/18:0, 20:0, and 24:1 were higher in the serum of genotype 1 HCV than hepatitis B virus (HBV)-infected patients. On the other hand, ceramide d18:1;O2/16:0 and 24:0 were reduced [16]. Whereas, all of these ceramide species did not differ in patients with mild and advanced fibrosis, ceramide d18:1;O2/24:0 declined in serum of patients with fibrosis stages 3 or 4. In multivariate analysis, none of these ceramide species in serum were associated with liver fibrosis stage, which was histologically diagnosed [16]. In the HCV patients, ceramide d18:1;O2/16:0, 18:0, 20:0, 24:0, and 24:1 generally did not correlate with aminotransferase levels and were not associated with the homeostatic model assessment index as a measure of insulin resistance [16].

Serum ceramide d18:1;O2/24:0 was about 10-fold lower in patients with liver cirrhosis compared to healthy controls. Additionally, ceramide d18:1;O2/18:0, 20:0, 24:0, and 24:1 declined with an increasing Child–Pugh score [18]. This was most evident for ceramide d18:1;O2/24:0, and a negative correlation with the model for end-stage liver disease (MELD) score existed [18]. Serum levels of lipoproteins were not available in this study cohort, and associations with LDL could not be calculated [18].

In liver cirrhosis, LDL is low and further declines with progression of liver disease [19]. Negative correlations between LDL and the MELD score were found in patients with liver cirrhosis [20]. The MELD score and LDL were predictors of survival in patients with decompensated liver cirrhosis [21,22]. Of clinical relevance, serum ceramide d18:1;O2/24:0 predicted survival of patients with liver cirrhosis as good as the MELD score [18].

Ceramide d18:1;O2/16:0, 18:0, 20:0, 24:0, and 24:1 were, however, not related to viral load [16]. Clinically relevant for treatment, ceramide d18:1;O2/18:1, 24:0, and 24:1 levels predicted sustained virologic response (SVR) to pegylated interferon alpha and ribavirin therapy [16].

The biologic activities of ceramides depend on the acyl-chain length of their fatty acids. Long-chain ceramides (C16–C20) contributed to insulin resistance and induced cell death, whereas very-long-chain ceramides (C22–C24) had the opposite effect [13,23,24]. Increased systemic levels of ceramide species were described in the obese, in insulin resistant patients, and in patients with cardiovascular disease [25]. In a community-based study, the plasma ceramide d18:1;O2/C24:0/C16:0 and the ceramide d18:1;O2/C22:0/C16:0 ratios were negatively associated with mortality. A high ceramide d18:1;O2/C24:0/C16:0 ratio was related to a lower risk for coronary heart disease [26].

Direct acting antiviral (DAA) therapy is highly effective in curing HCV [27] and is well-described to raise serum LDL levels [6,7,28]. Unexpectedly, ceramide d18:1;O2/16:0, 18:0, and 24:1 were reduced at sustained virologic response at 12 weeks post-treatment (SVR12) with DAAs, whereas ceramide d18:1;O2/22:0 increased [29].

Current evidence suggests that serum ceramides are associated with markers of liver injury in patients with chronic HCV infection. To study this in more depth, levels of ceramide d18:1;O2/16:0, 18:0, 20:0, 22:0, 23:0, 24:0, 24:1, and 26:0 were analyzed in the sera of 178 patients with chronic HCV infection. Associations with measures of liver function were calculated in the entire cohort and in the subgroups of cirrhosis and non-cirrhosis patients, before and at 12 weeks after the start of DAA therapy.

## 2. Results

### 2.1. Association of Ceramide Species Levels with Gender, Fatty Liver, Diabetes, Age, and Body Mass Index in HCV Patients

Ceramides are multifunctional lipids that have a central role in chronic liver diseases [13,14,15,16]. Here, ceramide d18:1;O2/16:0, 18:0, 20:0, 22:0, 23:0, 24:0, 24:1, and 26:0 were measured in the sera of 178 patients with chronic HCV infection (Table 1).

**Figure 1:**
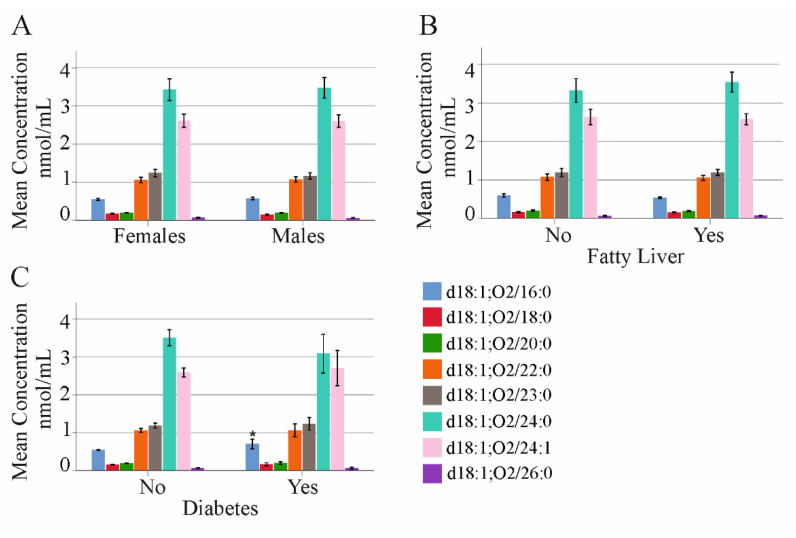
Ceramide species in relation to gender, fatty liver disease, and diabetes in patients with chronic HCV. (**A**) Ceramide species in 74 female and 104 male patients; (**B**) ceramide species in 104 patients without (No) and 74 patients with (Yes) fatty liver disease; (**C**) ceramide species in 154 patients without (No) and 20 patients with (Yes) diabetes. Mean concentration ± 95% confidence interval is shown. * *p* < 0.05.

Levels of these ceramide species did not differ between the 104 male and the 74 female patients (Figure 1A). Seventy-four patients had fatty liver disease, which was not associated with higher concentrations of serum ceramides (Figure 1B). Compared to the non-diabetic patients, ceramide d18:1;O2/16:0 was higher in the serum of the 20 diabetic patients (Figure 1C). Moreover, ceramide d18:1;O2/24:0 was negatively correlated with age (Table 2). Ceramide levels were not associated with body mass index (BMI) (Table 2).

### 2.2. Ceramide Species Levels and the Model for End-Stage Liver Disease Score in HCV Patients before DAA Therapy

Previous studies described significant negative associations of serum ceramide d18:1;O2/24:0 levels with liver fibrosis stage and the model for end-stage liver disease (MELD) score [16,18].

In our 178 patients with HCV infection, ceramide d18:1;O2/24:0 was negatively correlated with the MELD score. Notably, ceramide d18:1;O2/18:0, 20:0, 22:0, 23:0, 24:0, 24:1, and 26:0 were all negatively associated with the MELD score (Table 2). When adjusted for LDL levels, the association of ceramide d18:1;O2/20:0 (*p* = 0.029), 22:0 (*p* = 0.01) and 24:0 (*p* < 0.001) was still significant. Bilirubin, creatinine, and international normalized ratio (INR) are used for MELD score calculation [30]. These ceramide species were negatively correlated with the INR and bilirubin (except ceramide d18:1;O2/26:0, which was not associated with bilirubin levels). Ceramide species did not correlate with creatinine (Table 2).

In line with these results, ceramide d18:1;O2/18:0, 20:0, 22:0, 23:0, 24:0, and 26:0 were positively correlated with albumin. Negative associations of ceramide d18:1;O2/20:0, 22:0, 23:0, and 24:0 with aspartate aminotransferase (AST) were found (Table 2). Ceramide d18:1;O2/20:0 and 24:1 positively correlated with leukocyte count (Table 2).

Ceramide d18:1;O2/16:0 did not correlate with the MELD score. It was negatively associated with the platelet count and positively with C-reactive protein (CRP). Ceramide d18:1;O2/18:0, 20:0, 22:0, 23:0, 24:0, 24:1, and 26:0 levels were not related with CRP, though were positively correlated with platelet count, showing that ceramide d18:1;O2/16:0 behaves differently from other ceramide species (Table 2).

All ceramides were positively correlated with LDL, whereas associations with HDL did not exist (Table 2 and Figure S1).

### 2.3. Ceramide Species Levels and MELD Score in HCV Patients at 12 Weeks after the Start of Therapy

DAA therapy efficiently eliminated HCV [7,31] and ferritin, ALT and aspartate aminotransferase (AST) declined (Table 1). Patients were treated with DAAs for 12 weeks, and at this time point all but ceramide d18:1;O2/16:0 and 24:1 were negatively correlated with the MELD score (Table S1). Ceramide d18:1;O2/24:0 was the only species that was significantly correlated with the MELD score, after adjusting for LDL levels (*p* = 0.016). The ceramide species d18:1;O2/22:0, 23:0, and 24:0 were negatively correlated with the INR. All but ceramide d18:1;O2/16:0 were negatively related to bilirubin and positively related to albumin. Ceramide d18:1;O2/20:0, 22:0, 23:0, 24:0, and 26:0 were negatively correlated with AST (Table S1). Leukocyte count was positively linked with ceramide d18:1;O2/20:0, 22:0, 24:0, and 24:1, and platelet number was positively linked with ceramide d18:1;O2/20:0, 22:0, 23:0 24:0, 24:1, and 26:0 (Table S1).

### 2.4. Ceramide Species Levels and Non-Invasive Scores of Liver Fibrosis/Cirrhosis in HCV Patients

**Figure 2:**
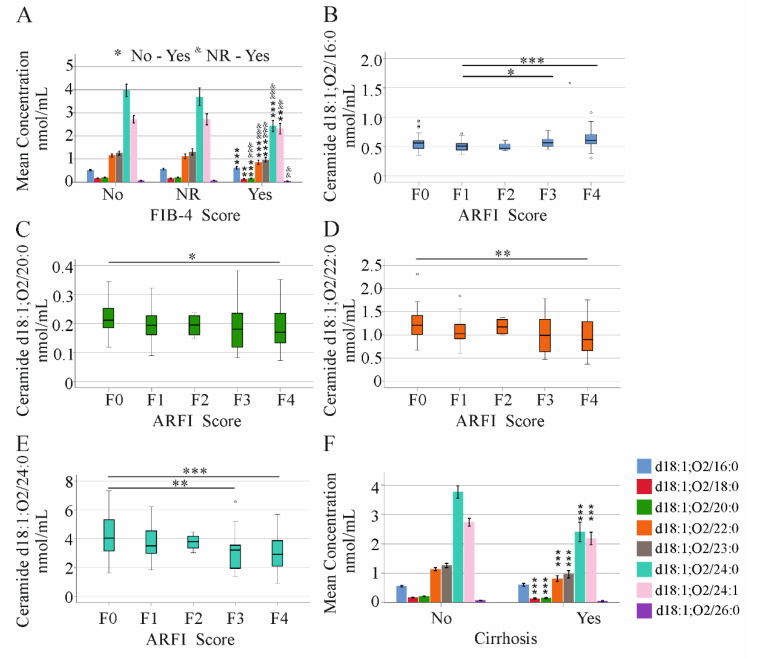
Ceramide species in relation to non-invasive fibrosis scores and liver cirrhosis diagnosed by ultrasound. (**A**) Mean concentration ± 95% confidence interval of ceramide levels in patients stratified for fibrosis by the FIB-4 score, (no fibrosis = No, 76 patients; not reliable values = NR, 52 patients; fibrosis = Yes, 50 patients); (**B**) median levels of ceramide d18:1;O2/16:0, (**C**) 20:0, (**D**) 22:0, and (**E**) 24:0 stratified for fibrosis by ARFI score (F0 = 69, F1 = 37, F2 = 11, F3 = 22, and F4 = 39 patients); (**F**) mean concentration ± 95% confidence interval of ceramide species in 138 patients without (No) and 40 patients with (Yes) liver cirrhosis diagnosed by ultrasound. * *p* < 0.05, ** *p* < 0.01, *** *p* < 0.001, ^&^ *p* < 0.05, ^&&^ *p* < 0.01, ^&&&^ *p* < 0.001.

The fibrosis-4 index correctly classifies HCV patients without liver fibrosis and patients with advanced fibrosis, but it cannot accurately diagnose intermediate fibrosis stages [32]. Ceramide d18:1;O2/18:0, 20:0, 22:0, 23:0, 24:0, and 24:1 were all reduced in patients with high FIB-4 scores, in comparison to patients without liver fibrosis (Figure 2A). Notably, ceramide d18:1;O2/16:0 was induced (Figure 2A). This applies to the patients before therapy start and at 12 weeks after treatment initiation (Figure 2A and Figure S2A).

Acoustic Radiation Force Impulse (ARFI) elastography is a method for non-invasive staging of advanced liver fibrosis [33,34]. ARFI values were obtained before therapy start. Here, ceramide d18:1;O2/16:0 was higher in patients with fibrosis stage 3 or 4, compared to patients with F1 scores (Figure 2B). Ceramide d18:1;O2/20:0, 22:0, and 24:0 were reduced in patients with cirrhosis, compared to patients with stage F0 (Figure 2C–E).

Along the same lines, all but ceramide d18:1;O2/16:0 and 26:0 were reduced in the serum of the 40 patients who were diagnosed with liver cirrhosis by ultrasound (Figure 2F). This did not change at 12 weeks after the start of therapy, though ceramide d18:1;O2/26:0 was also low in cirrhosis patients at this later time point (Figure S2B).

It is important and of clinical relevance to note that LDL was low in liver cirrhosis patients (*p* < 0.001), whereas HDL was similar in patients with and without cirrhosis at both time points (*p* > 0.05).

### 2.5. Ceramide Species Levels and MELD Score in HCV Patients with and without Liver Cirrhosis

Having shown the decline of most ceramides in liver cirrhosis patients (Figure 2F), we calculated correlations with measures of liver disease separately in cirrhosis and non-cirrhosis patients.

In the 138 untreated HCV patients without liver cirrhosis, ceramide species did not change in the 8 patients with diabetes (*p* > 0.05) or in the 53 patients with fatty liver disease (*p* > 0.05) and was similar in the 58 females and 80 males (*p* > 0.05). Ceramide levels were not associated with BMI, and only ceramide d18:1;O2/16:0 was positively correlated with age (Table 3).

In the HCV patients not suffering from liver cirrhosis, none of the ceramide species were correlated with the MELD score (Table 3). Ceramide d18:1;O2/24:0 was negatively correlated with AST and INR, and ceramide d18:1;O2/16:0 and 24:1 were positively related to CRP (Table 3). All species were positively linked with LDL but not HDL (Table 3).

**Figure 3:**
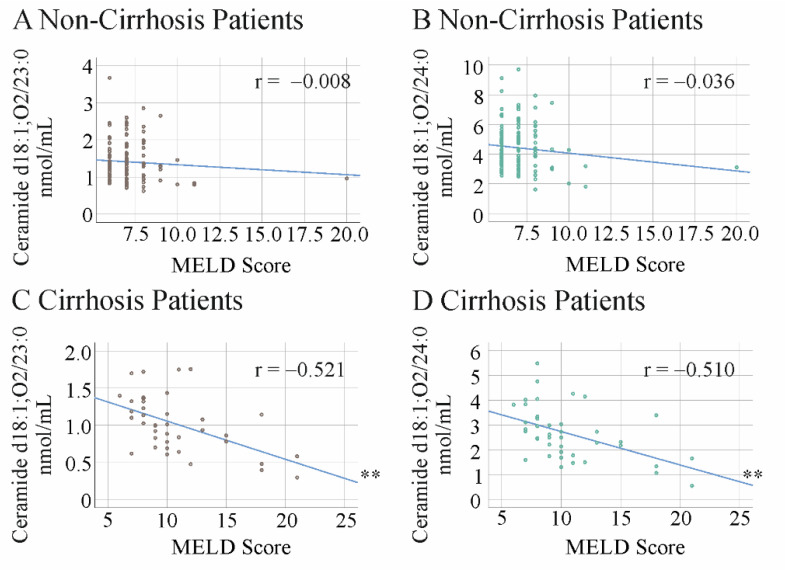
Correlation of ceramide d18:1;O2/23:0 and 24:0 with the MELD score at therapy end. (**A**) Correlation of ceramide d18:1;O2/23:0 with the MELD score in non-cirrhosis patients; (**B**) correlation of ceramide d18:1;O2/24:0 with the MELD score in non-cirrhosis patients; (**C**) correlation of ceramide d18:1;O2/23:0 with the MELD score in cirrhosis patients; (**D**) correlation of ceramide d18:1;O2/24:0 with the MELD score in cirrhosis patients. ** *p* < 0.01.

At the end of therapy, no significant correlations between the ceramide species and the MELD score were observed (Figure 3A,B and Table S2). Ceramide d18:1;O2/23:0 was positively correlated with age, and ceramide d18:1;O2/20:0, 23:0, and 24:1 were negatively correlated with bilirubin (Table S2).

In the group of 40 patients with liver cirrhosis, the 16 females and 24 males had similar serum levels of these ceramide species (*p* > 0.05). Diabetes (12 patients, *p* > 0.05) and fatty liver disease (21 patients, *p* > 0.05) were not linked to changes of any ceramide variant. In the patients with liver cirrhosis, none of the ceramides were associated with the MELD score before the start of DAA therapy. Positive correlations of ceramide d18:1;O2/22:0, 23:0, and 24:0 and albumin were observed (Table 4). Ceramide d18:1;O2/20:0, 23:0, 24:0, and 24:1 were negatively correlated with INR. Positive correlations between ceramide d18:1;O2/26:0 and leukocyte count and ceramide d18:1;O2/24:0 and 26:0 and platelet number were also identified (Table 4).

None of the ceramides were correlated with HDL levels, and ceramide d18:1;O2/20:0, 22:0, 23:0. 24:0, and 24:1 were positively associated with LDL levels (Table 4).

At the end of therapy, ceramide d18:1;O2/23:0 and 24:0 were negatively associated with the MELD score (Figure 3C,D and Table S3). Notably, when adjusted for LDL levels, the correlation of ceramide d18:1;O2/23:0 with the MELD score was still significant (*p* = 0.048). Ceramide d18:1;O2/23:0 was also negatively correlated with bilirubin, whereas d18:1;O2/24:0 was positively correlated with platelet count. All but ceramide d18:1;O2/16:0, 18:0, and 26:0 were negatively correlated with INR (Table S3).

### 2.6. Impact of DAA Therapy on Serum LDL and Ceramide Levels

**Figure 4:**
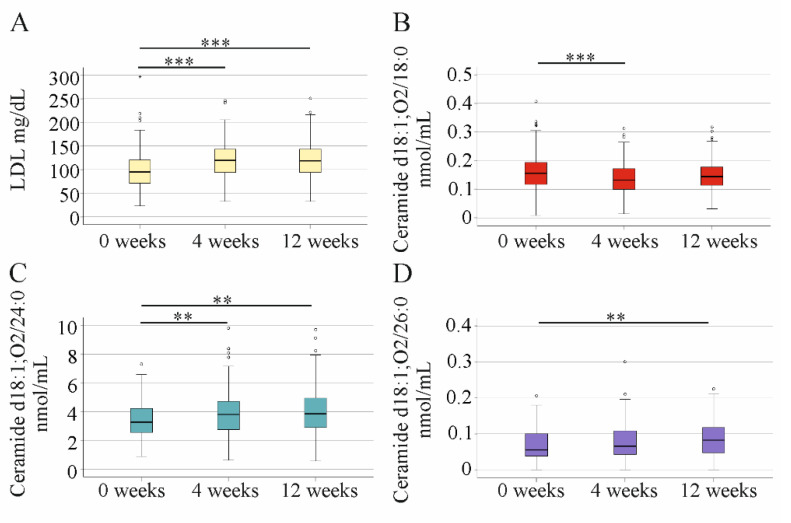
LDL and ceramide levels during the study. (**A**) LDL; (**B**) ceramide d18:1;O2/18:0; (**C**) ceramide d18:1;O2/24:0; and (**D**) ceramide d18:1;O2/26:0 before therapy (0 weeks; 178 patients) and at 4 weeks (178 patients) and 12 weeks (176 patients) after start of treatment. ** *p* < 0.01, *** *p* < 0.001.

Consistent evidence has shown that LDL, which is low in HCV, strongly increased after 4 weeks DAA therapy [6,35,36] (Figure 4A). Ceramide d18:1;O2/18:0 declined within 4 weeks of therapy and did not further change at 12 weeks. An increase in ceramide d18:1;O2/24:0 was detected at 4 weeks after the start of DAA therapy. Ceramide d18:1;O2/26:0 was induced at the end of treatment, when compared to the levels before therapy (Figure 4D). HDL concentration in serum was 52 (19–111) mg/dL before therapy, 54 (22–102) mg/dL at week 4, and 50 (13–96) mg/dL at week 12, so did not change during treatment.

### 2.7. Very-Long-Chain to Long-Chain Ceramides in HCV Patients before Therapy and at 12 Weeks after Start of Treatment

**Figure 5:**
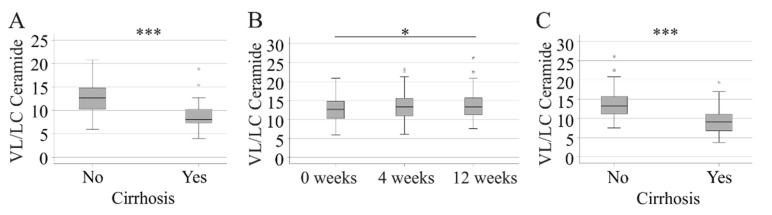
Very-long-chain to long-chain ceramide ratio (VL/LC). (**A**) VL/LC ceramide in 138 patients without and 40 patients with liver cirrhosis before the start of therapy; (**B**) VL/LC ceramide in patients without liver cirrhosis during the study. Data at 0 and 4 weeks for 138 patients and data at 12 weeks for 136 patients are shown; (**C**) VL/LC ceramide in 136 patients without and 40 patients with liver cirrhosis at week 12. * *p* < 0.05, *** *p* < 0.001.

Convincing evidence showed that the ratio of very-long-chain (VL) to long-chain (LC) ceramides, rather than the absolute levels of these lipids, is associated with different diseases and overall mortality [26]. This ratio was higher in the HCV patients without liver cirrhosis before the start of therapy (Figure 5A). An increase at week 12, compared to levels before DAA therapy, was detected in the non-cirrhosis patients (Figure 5B). Accordingly, the VL/LC ratio was higher in the non-cirrhosis patients than in the cirrhosis patients at week 12 (Figure 5C).

The VL/LC ratio was similar in patients with and without fatty liver disease (*p* > 0.05 at 0 and at 12 weeks). Patients with diabetes had a lower value at week 0 (*p* = 0.006) but not at week 12 (*p* = 0.134). Moreover, this ratio did not correlate with the MELD score in patients with and without liver cirrhosis at any time point.

### 2.8. Association of Ceramide Species Levels with Viral Load and Genotype in Non-Cirrhosis HCV Patients

A positive association of ceramide d18:1;O2/20:0 with viral load was detected in the whole cohort before DAA therapy (Table 2). In the subgroups of HCV patients without or with liver cirrhosis, correlations of viral load and ceramide species were not found (Table 3 and Table 4).

**Figure 6:**
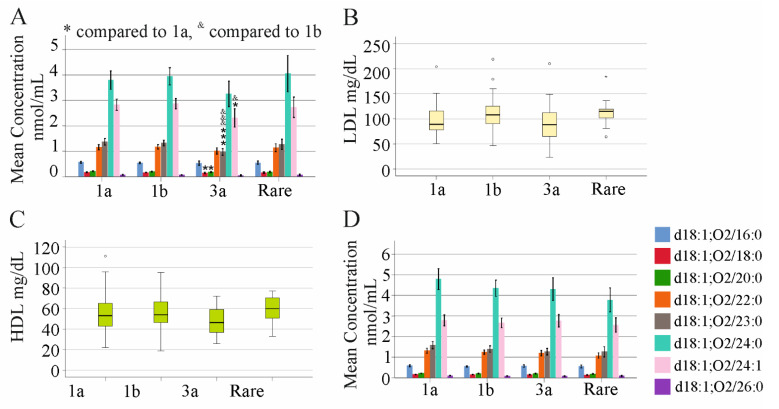
Ceramide levels stratified for viral genotypes. (**A**) Ceramides in serum of patients with different viral genotypes before therapy start; (**B**) LDL and (**C**) HDL levels of these patients; (**D**) ceramides in serum of patients with different viral genotypes at the end of therapy. * *p* < 0.05, *** *p* < 0.001, ^&^ *p* < 0.05, ^&&&^ *p* < 0.001.

Based on the observed decrease of most serum ceramide species in patients with liver cirrhosis, associations with viral genotypes were calculated in HCV patients without liver cirrhosis. Here, 43 patients had genotype 1a, 53 patients had genotype 1b, 29 patients had genotype 3a, and 13 patients had rare genotypes such as 2 or 2a. Ceramide d18:1;O2/18:0, 20:0, 23:0, and 24:1 were lower in genotype-3a-infected patients than genotype-1a-infected patients. Ceramide d18:1;O2/23:0 and 24:1 were lower in genotype-3a-infected patients compared to genotype-1b-infected patients (Figure 6A). The VL/LC ratio was, however, similar between the genotypes (*p* > 0.05). LDL and HDL levels did not vary between the genotypes (Figure 6B,C). Prevalence of liver steatosis was 59%, 51%, 48% and 61% in genotype 1a, 1b, 3a, and the group with rare genotypes, respectively, and was similar between all groups (*p* > 0.05). At 12 weeks post therapy initiation, ceramide species did not differ between the genotypes (Figure 6D).

## 3. Discussion

Here, we show that serum ceramide d18:1;O2/18:0, 20:0, 22:0, 23:0, 24:0, 24:1, and 26:0 levels are low in HCV patients with liver cirrhosis. Ceramide d18:1;O2/16:0 is comparable in patients with and without cirrhosis, and this contributes to an adverse ceramide profile of HCV patients with liver cirrhosis. DAA therapy causes a more favorable ceramide species composition in the serum of patients without liver cirrhosis. Such an effect does not occur in cirrhosis patients. Notably, the more beneficial ceramide species, d18:1;O2/23:0 and 24:0, were negatively correlated with the MELD score in patients with liver cirrhosis at the end of DAA therapy.

Ceramide species’ levels in HCV cirrhosis have been studied by Grammatikos et al. [16]. Their investigation showed that ceramide d18:1;O2/24:0 was lower in F3/4 fibrosis (which applied to 25% of the 203 genotype-1-infected HCV patients enrolled in this study) than in F0–F2 fibrosis. A decrease of ceramide d18:0/18:0, 20:0, and 24.1 in liver cirrhosis was not observed. Moreover, in multivariate analysis the association of ceramide d18:1;O2/24:0 with the liver fibrosis stage was no longer significant. The liver fibrosis stage was histologically evaluated in the study by Grammatikos et al. [16].

In our investigation, ultrasound and the non-invasive FIB-4 and ARFI scores were used. FIB-4-defined liver cirrhosis as well as ultrasound-diagnosed cirrhosis were linked with low levels of all of the analyzed ceramides, except ceramide d18:1;O2/16:0. This latter variant was induced in liver cirrhosis, as assessed by FIB-4, and was unchanged in ultrasound-diagnosed cirrhosis. Cirrhosis defined by ARFI was associated with low ceramide d18:1;O2/20:0, 22:0, and 24:0 levels. It has to be emphasized that ARFI levels not only reflect fibrosis but also are associated with viral inflammation and, accordingly, decline after effective elimination of HCV [31]. None of the ceramide species were reduced in patients with intermediate FIB-4 or ARFI scores. In summary, this shows that ceramides do not decline in parallel with increasing fibrosis stages. Thus, grouping patients with F3 and F4 fibrosis in one cohort, as was done in the study of Grammatikos et al., may fail to identify low levels of ceramides in F4 fibrosis [16].

Negative associations of all ceramides, except ceramide d18:1;O2/16:0, with the MELD score were identified in the current study cohort before therapy. All, except ceramide d18:1;O2/16:1 and 24:1, were negatively associated with the MELD score at the end of therapy. When analysis was conducted separately in untreated non-cirrhosis and cirrhosis patients, no such correlations were identified. At 12 weeks after the start of therapy, a negative correlation between the MELD score and ceramide d18:1;O2/23:0 and 24:0 was observed in the patients with liver cirrhosis. Correlations of ceramide d18:1;O2/23:0 and 24:0 with the MELD score were described before in patients with alcoholic liver cirrhosis. Ceramide d18:1;O2/16:0, 18:0, 20:0, 22:0, and 24:1 did not, however, show an association with the MELD score in patients with alcohol abuse as an underlying cause of liver cirrhosis [37]. In 244 patients with liver cirrhosis of different etiologies, ceramide d18:1;O2/18:0, 20:0, 24:0, and 24:1 declined with an increasing Child–Pugh score, which was most evident in HCV patients. In this cohort, ceramide d18:1;O2/24:0 was negatively correlated with the MELD score [18]. Thus, ceramide d18:1;O2/24:0 is negatively linked with the MELD score in patients with liver cirrhosis of different etiologies.

Ceramides are carried in LDL particles [12]. When adjusted for LDL levels, the correlation of the MELD score and ceramide d18:1;O2/23:0 in our HCV patients with liver cirrhosis was still significant at therapy end. This revealed that ceramide d18:1;O2/23:0 is associated with the MELD score independently of LDL, which also declines in liver cirrhosis patients [3].

Correlations of ceramide species with the laboratory parameters of liver disease severity were found in the entire cohort, although not when cirrhosis and non-cirrhosis patients were separately analyzed. This shows that the observed correlations with indicators of liver dysfunction are not linear but reflect the differences between the HCV patients with and without liver cirrhosis.

LDL carries a significant amount of serum ceramides, and LDL is well-described as increasing shortly after the start of DAA therapy [6,7,12,28,36]. Whereas ceramide d18:1;O2/24:0 increased in parallel, ceramide d18:1;O2/26:0 was only elevated at the end of therapy. Ceramide d18:1;O2/18:0 even declined within 4 weeks of treatment. From this, it can be concluded that efficient elimination of HCV not only raises LDL and LDL-associated lipids but alters the ceramide composition of the serum and probably LDL during therapy. Likewise, it was shown that the VL/LC ratio increases during DAA treatment. Thus, removal of HCV clearly produces a more favorable ceramide serum profile.

In non-treated patients with genotype 3 infection, ceramide d18:1;O2/18:0, 20:0, 23:0, and 24:1 were low compared to genotype-1-infected patients. The VL/LC ceramide ratio and LDL levels were similar between the genotypes. Genotype 3 was associated with liver steatosis and a more rapid progression of liver fibrosis [38]. Whether lower levels of almost all ceramides play a role in fibrosis progression needs further study. At the end of therapy, the genotype-related differences of the ceramides disappeared, suggesting a stronger effect of genotype 3 infection than genotype 1 infection on ceramide metabolism. The changed ceramide levels of these patients are seemingly not related to liver steatosis, because the serum ceramides did not change in patients with fatty liver disease, and this holds for the whole cohort before and during therapy and when patients were stratified for cirrhosis. Moreover, prevalence of fatty liver disease and LDL levels did not vary between the genotypes in our cohort.

DAA therapy is highly effective, and SVR12 was achieved in all but one patient in our cohort. In patients treated with pegylated interferon alpha/ribavirin, ceramide d18:1;O2/24:0, 24:1, and 18:1 were found positively associated with SVR [16]. Pegylated interferon alpha/ribavirin therapy is less effective in patients with liver cirrhosis [39,40,41]. According to the data shown in this work and studies published by other researchers [13,16], patients with liver cirrhosis have lower serum ceramide levels. Thus, the association of low serum ceramides with treatment failure may be because these patients suffer from cirrhosis.

Ceramide d18:1;O2/16:0 behaves differently than the other ceramide species measured in our study. This lipid species did not correlate with the MELD score and was not reduced in the serum of patients with liver cirrhosis. Current results suggest that ceramide d18:1;O2/16:0 in serum is not related to liver dysfunction in cirrhosis. Notably, ceramide d18:1;O2/16:0 is nearly two-fold more abundant in HDL than LDL [12]. Similarly to ceramide d18:1;O2/16:0, HDL was not reduced in liver cirrhosis. Whether serum ceramide d18:1;O2/16:0 mostly corresponds to the levels in HDL, thus, needs further analysis.

Ceramide d18:1;O2/16:0 was, however, positively correlated with LDL but not HDL. This was in accordance with all of the ceramides analyzed. Low levels of LDL and ceramides in liver cirrhosis strongly suggest that impaired hepatic VLDL release and reduced hepatic lipid levels are the underlying condition. The liver seems to be the predominant organ for production and reuptake of serum ceramides [25]. Yet, adipose tissue, skeletal muscle, and the gut may also affect serum ceramide levels [25]. Future research will have to identify the main organs and pathways regulating systemic ceramide levels.

Ceramide d18:1;O2/16:0 was linked to insulin resistance, diabetes, and cardiovascular diseases [25]. In the HCV cohort, diabetes patients had higher levels of ceramide d18:1;O2/16:0 before therapy but not at the end of therapy. Indeed, all of the other measured ceramides did not change in patients with diabetes. This illustrates that serum ceramides are unsuitable biomarkers for diabetes, at least in HCV-infected patients.

One important question is whether low ceramide serum levels are just indicators of impaired hepatic synthesis or have a role in liver function. At least the current analysis could not identify strong associations of serum ceramide levels and markers of hepatic dysfunction. A limitation of our study is that the liver lipids could not be analyzed.

Most studies published so far have investigated the effects of lowering high ceramide levels, which are associated with metabolic diseases and non-alcoholic fatty liver disease [42]. Myriocin inhibits serine palmitoyltransferase, which catalyzes the first reaction of sphingolipid biosynthesis. Rats chronically treated with this drug had about 50% lower serum ceramide, and this was associated with improved liver steatosis and fibrosis [43].

The median serum ceramide levels of our patients with liver cirrhosis were about 25% lower than those of patients without cirrhosis. Various other lipids and proteins are dysregulated in patients with liver cirrhosis [19,20,44,45,46], and all of these changes may finally contribute to disease severity and prognosis. There is, however, evidence that relatively small variations of ceramide levels are of pathophysiological relevance. In muscle tissues of type 2 diabetic patients, an approximately 20% higher concentration of long-chain ceramides was detected. Animal studies showed that about a 30% lower level of these ceramides in skeletal muscle improved the unfolded protein response [47]. Liver-specific overexpression of acid ceramidase lowered alcoholic liver steatosis and hepatic insulin resistance, though hepatic ceramide species only modestly declined [48].

Tissue ceramide levels are approximately 20% lower in hepatocellular carcinoma (HCC) than in non-cancer tissue. It is supposed that this relatively small difference in ceramide concentration contributes to tumor growth [13,42,49].

Suggesting that low serum ceramide is related to low hepatic ceramide levels in patients with liver cirrhosis, a possible association with cancer development may exist. Grammatikos et al. showed an upregulation of serum C16 ceramide in patients with HCC [50]. In this cohort, patients without HCC had worse liver function than the HCC patients. Because C16 ceramide is not changed in cirrhosis patients, this difference in liver function may not account for the higher serum C16 ceramide levels in HCC. Therefore, further studies have to prove an association between serum ceramides and HCC.

Previous studies revealed that ceramide species’ ratios, rather than the levels of individual species, may serve as biomarkers for different diseases [13,25]. The VL/LC ceramide ratio did not correlate with the MELD score but was reduced in the serum of our patients with liver cirrhosis. Mice deficient in ceramide synthase 2 are unable to synthesize very-long-chain ceramides and suffer from hepatic insulin resistance [51]. Higher hepatic insulin resistance is relatively common in patients with liver cirrhosis [52,53], and the adverse ceramide composition may have a role herein.

Future research has to study the hepatic effects of ceramide species disequilibrium in liver cirrhosis.

## 4. Materials and Methods

### 4.1. Study Cohort

This study was conducted at the Department of Internal Medicine I at the University Hospital of Regensburg from October 2014 to September 2019 [31]. All patients appropriate for DAA therapy, according to recent guidelines [54], were enrolled in the study. Patients signed an informed consent when they agreed to participate. HCV treatment naive patients were treated with DAAs for 12 weeks. All patients were older than 18 years. Patients were not coinfected with HBV or human immunodeficiency virus.

All patients had chronic HCV and were treated with one of the following regimens: sofosbuvir/daclatasvir (74), sofosbuvir/daclatasvir/ribavirin (4), sofosbuvir/ledipasvir (47), sofosbuvir/ledipasvir/ribavirin (7), sofosbuvir/velpatasvir (6), sofosbuvir/ribavirin (10), or dasabuvir/ombitasvir/paritaprevir/ritonavir (30), in accordance with international treatment guidelines [54]. Laboratory parameters were obtained from the Institute of Clinical Chemistry and Laboratory Medicine (University Hospital Regensburg).

There were only very few patients using statins, so statins were paused during treatment.

Cirrhosis diagnosis was established by ultrasound and liver surface, and size of the liver and liver parenchyma were analyzed [55]. Cut-off values used for the FIB-4 scoring were the following: advanced fibrosis > 3.25, no fibrosis < 1.30 for patients younger than 65 years and <2.00 for patients older than 65 years [56]. Patients’ characteristics are given in Table 1. Laboratory values were not always available from all patients, and data of at least 95% of the patients were included.

### 4.2. Lipid Extraction and Analysis of Ceramide Species

Internal standards (Cer 18:1;O2/14:0 and Cer 18:1;O2/17:0; Avanti Polar Lipids, Alabaster, AL, USA) were added and vacuum-dried prior to extraction. A volume of 10 µL serum was subjected to lipid extraction, according to the protocol described by Bligh and Dyer [57], with a total chloroform volume of 2 mL. An amount of 1 mL of the separated chloroform phase was transferred into sample vials by a pipetting robot (Tecan Genesis RSP 150, Männedorf, Switzerland) and vacuum-dried. The residue was dissolved in methanol (Merck, Darmstadt, Germany)/chloroform (Roth, Karlsruhe, Germany) (3:1, *v*/*v*) with 7.5 mM ammonium acetate (FIA-MS/MS) [58].

The analysis of lipids was performed by direct flow injection analysis (FIA) using a triple quadrupole mass spectrometer (FIA-MS/MS; QQQ triple quadrupole). The measurement was performed in positive ion mode. Sphingosine-based Cer were analyzed using a fragment ion of *m*/*z* 264. Quantification was performed with calibration lines, as previously described in detail [59].

### 4.3. Statistical Analysis

Data are shown as boxplots, which gives the minimum, the maximum, the median, and the first and third quartiles. Small circles or asterisks above or below the boxes mark outliers. When more than one ceramide species was displayed in a figure, data are shown as mean concentration ± 95% confidence interval.

Data are reported as median values, and the minimum and maximum values are given in brackets.

The non-parametric Kruskal–Wallis test was used for comparison of continuous variables between independent groups. Student’s t-test was used to analyze paired data, and Spearman’s correlation was used for correlation analysis (SPSS Statistics 26.0 program, IBM, Armonk, NY, USA). Chi-squared test (MS Excel, Microsoft Co., Ltd., Albuquerque, NM, USA) was used for categorical variables. Data were corrected for multiple comparisons. A value of *p* < 0.05 was regarded as significant.

## 5. Conclusions

The current study showed that the serum ceramide profile is changed by HCV infection and the HCV genotype. Moreover, serum ceramides are low in liver cirrhosis and have an unfavorable ceramide species profile. Thus, serum ceramide species should be further evaluated as potential markers for the insulin resistance, HCC development, and prognosis of liver cirrhosis.

## Figures and Tables

**Table 1:** Laboratory parameters of the patients at baseline and after 12 weeks of antiviral therapy (alanine amino transferase (ALT), aspartate aminotransferase (AST), body mass index (BMI), C-reactive protein (CRP), high-density lipoprotein (HDL), international normalized ratio (INR), low-density lipoprotein (LDL), model of end-stage liver disease (MELD), not significant (ns)).

Laboratory Parameter	Baseline (178 Patients)	12 Weeks Therapy (176 Patients)	*p*-Value
Age, years	54 (24–82)	54 (24–82)	ns
BMI, kg/m^2^	25.6 (17.6–41.6)	25.6 (17.6–41.6)	ns
MELD	7 (6–21)	7 (6–21)	ns
Platelets, n/nL	195 (38–402)	206 (37–407)	ns
Ferritin, ng/mL	128.6 (5.6–2309)	94.2 (2.9–1161)	0.003
ALT, U/L	61 (2–305)	26 (6–388)	<0.001
AST, U/L	47 (7–1230)	22 (6–836)	<0.001
Bilirubin, mg/dL	1.0 (1.0–4.3)	1.0 (1.0–7.5)	ns
Albumin, g/L	38 (2–50)	39 (16–93)	ns
INR	1.05 (1.00–2.44)	1.04 (1.00–2.22)	ns
Creatinine, mg/dL	0.78 (0.14–14.00)	0.76 (0.14–14.7)	ns
Leukocytes, n/L	6.5 (2.2–72.4)	6.8 (2.4–62.9)	ns
CRP, mg/L	2.9 (1.0–55.0)	2.9 (2.9–20.3)	ns
HDL, mg/dL	52 (19–111)	50 (13–96)	ns
LDL, mg/dL	95 (23–296)	119 (33–251)	<0.001

**Table 2:** Spearman correlation coefficients and *p*-values for the correlations of ceramide species with BMI, age, MELD score, viral load, and routine laboratory parameters in the HCV patients before direct-acting antiviral (DAA) therapy. Significance level was set to *p* < 0.05. Significant correlations are in bold (alanine amino transferase (ALT), aspartate aminotransferase (AST), body mass index (BMI), C-reactive protein (CRP), high-density lipoprotein (HDL), international normalized ratio (INR), low-density lipoprotein (LDL), model of end-stage liver disease (MELD), not significant (ns)).

		Ceramide Species d18:1;O2/nmol/mL							
Variables		16:0	18:0	20:0	22:0	23:0	24:0	24:1	26:0
BMI, kg/m^2^	r	0.079	−0.052	0.032	0.118	0.121	0.023	0.013	−0.101
	*p*	-	-	-	-	-	-	-	-
Age, years	r	0.200	0.062	0.017	−0.136	−0.012	**−0.239**	−0.012	−0.022
	*p*	-	-	-	-	-	**0.011**	-	-
MELD score	r	0.124	**−0.317**	**−0.346**	**−0.380**	**−0.298**	**−0.461**	**−0.295**	**−0.205**
	*p*	-	**<0.001**	**<0.001**	**<0.001**	**<0.001**	**<0.001**	**<0.001**	**0.049**
ALT, U/L	r	0.117	−0.191	**−0.226**	**−0.286**	**−0.247**	**−0.352**	−0.176	−0.165
	*p*	-	-	**0.019**	**<0.001**	**0.007**	**<0.001**	-	-
AST, U/L	r	0.005	−0.156	−0.118	−0.164	−0.143	−0.151	−0.117	−0.115
	*p*	-	-	-	-	-	-	-	-
Bilirubin, mg/dL	r	0.085	**−0.213**	**−0.255**	**−0.257**	**−0.240**	**−0.322**	**−0.208**	−0.111
	*p*	-	**0.034**	**0.005**	**0.004**	**0.010**	**<0.001**	**0.043**	-
Albumin, g/L	r	−0.133	**0.248**	**0.279**	**0.309**	**0.228**	**0.368**	0.200	**0.212**
	*p*	-	**0.008**	**0.002**	**<0.001**	**0.021**	**<0.001**	-	**0.041**
INR	r	0.174	**−0.289**	**−0.320**	**−0.395**	**−0.336**	**−0.496**	**−0.308**	**−0.237**
	*p*	-	**<0.001**	**<0.001**	**<0.001**	**<0.001**	**<0.001**	**<0.001**	**0.011**
Creatinine, mg/dL	r	−0.069	−0.039	−0.093	−0.078	0.002	−0.046	−0.009	0.053
	*p*	-	-	-	-	-	-	-	-
Leukocytes, n/L	r	−0.066	0.201	**0.237**	0.121	0.127	0.201	**0.255**	0.090
	*p*	-	-	**0.011**	-	-	-	**0.004**	-
Platelets, n/nL	r	**−0.215**	**0.249**	**0.274**	**0.249**	**0.222**	**0.397**	**0.272**	**0.253**
	*p*	**0.031**	**0.006**	**0.002**	**0.007**	**0.023**	**<0.001**	**0.002**	**0.005**
CRP, mg/L	r	**0.233**	0.100	0.147	0.076	0.064	0.053	0.177	0.088
	*p*	**0.014**	-	-	-	-	-	-	-
Ferritin, ng/mL	r	0.053	0.029	0.049	0.030	0.023	−0.056	0.097	−0.028
	*p*	-	-	-	-	-	-	-	-
Viral load	r	−0.069	0.131	**0.208**	0.158	0.144	0.114	0.064	−0.009
	*p*	-	-	**0.043**	-	-	-	-	-
HDL, mg/dL	r	0.033	0.110	0.059	0.077	0.094	0.131	0.044	0.136
	*p*	-	-	-	-	-	-	-	-
LDL, mg/dL	r	**0.291**	**0.310**	**0.517**	**0.614**	**0.624**	**0.666**	**0.578**	**0.383**
	*p*	**0.001**	**<0.001**	**<0.001**	**<0.001**	**<0.001**	**<0.001**	**<0.001**	**<0.001**

**Table 3:** Spearman correlation coefficients and *p*-values for the correlations of ceramide species with BMI, age, MELD score, viral load, and routine laboratory parameters in the 138 HCV patients without liver cirrhosis before direct-acting antiviral (DAA) therapy. Significant correlations are in bold (alanine amino transferase (ALT), aspartate aminotransferase (AST), body mass index (BMI), C-reactive protein (CRP), high-density lipoprotein (HDL), international normalized ratio (INR), low-density lipoprotein (LDL), model of end-stage liver disease (MELD), not significant (ns)).

		Ceramide Species d18:1;O2/nmol/mL							
Variables		16:0	18:0	20:0	22:0	23:0	24:0	24:1	26:0
BMI, kg/m^2^	r	0.055	0.037	0.101	0.196	0.167	0.067	0.027	−0.092
	*p*	-	-	-	-	-	-	-	-
Age, years	r	**0.252**	0.183	0.187	0.025	0.152	−0.103	0.147	0.063
	*p*	**0.023**	-	-	-	-	-	-	-
MELD score	r	0.043	−0.150	−0.101	−0.191	−0.116	−0.230	−0.130	−0.080
	*p*	-	-	-	-	-	-	-	-
ALT, U/L	r	0.082	−0.148	−0.168	−0.205	−0.173	−0.194	−0.137	−0.134
	*p*	-	-	-	-	-	-	-	-
AST, U/L	r	−0.003	−0.105	−0.119	−0.168	−0.127	**−0.234**	−0.077	−0.119
	*p*	-	-	-	-	-	**0.046**	-	-
Bilirubin, mg/dL	r	−0.026	−0.058	−0.076	−0.046	−0.022	−0.024	−0.004	0.015
	*p*	-	-	-	-	-	-	-	-
Albumin, g/L	r	−0.069	0.115	0.027	0.082	0.007	0.123	0.018	0.067
	*p*	-	-	-	-	-	-	-	-
INR	r	0.104	−0.140	−0.072	−0.219	−0.148	**−0.286**	−0.145	−0.127
	*p*	-	-	-	-	-	**0.005**	-	-
Creatinine, mg/dL	r	−0.040	−0.031	−0.041	0.009	0.041	0.019	0.031	0.106
	*p*	-	-	-	-	-	-	-	-
Leukocytes, n/L	r	−0.008	0.152	0.127	−0.019	−0.044	0.001	0.140	−0.072
	*p*	-	-	-	-	-	-	-	-
Platelets, n/nL	r	−0.196	0.124	0.052	0.041	0.012	0.156	0.101	0.119
	*p*	-	-	-	-	-	-	-	-
CRP, mg/L	r	**0.260**	0.136	0.160	0.128	0.082	0.139	**0.258**	0.150
	*p*	**0.017**	-	-	-	-	-	**0.018**	
Ferritin, ng/mL	r	0.034	−0.017	−0.010	−0.023	0.001	−0.093	0.037	0.001
	*p*	-	-	-	-	-	-	-	-
Viral load	r	−0.031	0.064	0.134	0.100	0.115	0.009	0.028	−0.010
	*p*	-	-	-	-	-	-	-	-
HDL, mg/dL	r	−0.043	0.055	−0.061	−0.030	0.006	0.059	−0.062	0.107
	*p*	-	-	-	-	-	-	-	-
LDL, mg/dL	r	**0.407**	**0.290**	**0.499**	**0.598**	**0.584**	**0.605**	**0.539**	**0.413**
	*p*	**<0.001**	**0.007**	**<0.001**	**<0.001**	**<0.001**	**<0.001**	**<0.001**	**<0.001**

**Table 4:** Spearman correlation coefficients and *p*-values for the correlations of ceramide species with BMI, age, MELD score, viral load, and routine laboratory parameters in the 40 HCV patients with liver cirrhosis before direct-acting antiviral (DAA) therapy. Significant correlations are in bold (alanine amino transferase (ALT), aspartate aminotransferase (AST), body mass index (BMI), C-reactive protein (CRP), high-density lipoprotein (HDL), international normalized ratio (INR), low-density lipoprotein (LDL), model of end-stage liver disease (MELD), not significant (ns)).

		Ceramide Species d18:1;O2/nmol/mL							
Variables		16:0	18:0	20:0	22:0	23:0	24:0	24:1	26:0
BMI, kg/m^2^	r	0.073	−0.159	−0.031	0.167	0.181	0.202	0.113	−0.022
	*p*	-	-	-	-	-	-	-	-
Age, years	r	−0.218	0.083	0.084	−0.172	−0.149	−0.168	−0.081	0.003
	*p*	-	-	-	-	-	-	-	-
MELD score	r	0.032	−0.326	−0.390	−0.257	−0.287	−0.392	−0.345	−0.294
	*p*	-	-	-	-	-	-	-	-
ALT, U/L	r	0.017	−0.225	0.111	−0.017	0.006	−0.006	−0.015	−0.008
	*p*	-	-	-	-	-	-	-	-
AST, U/L	r	0.019	−0.209	<0.001	−0.242	−0.242	−0.271	−0.109	−0.039
	*p*	-	-	-	-	-	-	-	-
Bilirubin, mg/dL	r	0.066	−0.194	−0.172	−0.251	−0.308	−0.372	−0.240	−0.146
	*p*	-	-	-	-	-	-	-	-
Albumin, g/L	r	0.040	0.281	0.380	**0.427**	**0.454**	**0.531**	0.309	0.347
	*p*	-	-	-	**0.048**	**0.026**	**0.003**	-	-
INR	r	−0.008	−0.350	**−0.491**	−0.384	**−0.495**	**−0.586**	**−0.480**	−0.352
	*p*	-	-	**0.010**	-	**0.009**	**0.001**	**0.014**	-
Creatinine, mg/dL	r	−0.231	0.030	−0.078	−0.101	0.079	−0.007	0.018	0.072
	*p*	-	-	-	-	-	-	-	-
Leukocytes, n/L	r	−0.043	−0.009	0.176	0.177	0.357	0.402	0.297	**0.436**
	*p*	-	-	-	-	-	-	-	**0.040**
Platelets, n/nL	r	−0.119	−0.074	0.241	0.214	0.367	**0.443**	0.238	**0.445**
	*p*	-	-	-	-	-	**0.034**	-	**0.032**
CRP, mg/L	r	0.163	0.194	0.258	0.124	0.106	0.057	0.169	−0.019
	*p*	-	-	-	-	-	-	-	-
Ferritin, ng/mL	r	0.176	0.102	0.283	0.226	0.147	0.105	0.367	−0.140
	*p*	-	-	-	-	-	-	-	-
Viral load	r	−0.083	0.049	−0.006	−0.064	−0.072	−0.021	−0.150	−0.196
	*p*	-	-	-	-	-	-	-	-
HDL, mg/dL	r	0.433	0.243	0.391	0.340	0.286	0.301	0.279	0.122
	*p*	-	-	-	-	-	-	-	-
LDL, mg/dL	r	0.414	0.384	**0.430**	**0.615**	**0.654**	**0.674**	**0.588**	0.191
	*p*	-	-	**0.042**	**<0.001**	**<0.001**	**<0.001**	**0.001**	-

## Data Availability

Original data can be obtained from the corresponding author.

## Supplementary Materials

The following supporting information can be downloaded at: https://www.mdpi.com/article/10.3390/ijms23179806/s1.
