# Supplementary material for: Serum Ceramide Species Are Associated with Liver Cirrhosis and Viral Genotype in Patients with Hepatitis C Infection
Source: Int J Mol Sci. 2022 Aug 29;23(17):9806. doi: 10.3390/ijms23179806 (PMC9456360; doi:10.3390/ijms23179806)
Supplement: Supplementary file 1 [file ijms-23-09806-s001.zip › ijms-1870545-supplementary.pdf]

Table S1. Spearman correlation coefficients and p-values for the correlation of ceramide species with BMI, age, MELD score, and routine laboratory parameters in the HCV patients at therapy end. Significant correlations are in bold. – not significant (alanine amino transferase (ALT), aspartate aminotransferase (AST), body mass index (BMI), C-reactive protein (CRP), high-density lipoprotein (HDL), international normalized ratio (INR), low-density lipoprotein (LDL), model of end stage liver disease (MELD).

**Ceramide species d18:1;O2/ nmol/ml**		**16:0**	**18:0**	**20:0**	**22:0**	**23:0**	**24:0**	**24:1**	**26:0**
BMI kg/m²	r	0.054	0.020	0.007	0.026	0.040	-0.002	-0.059	-0.093
	p	-	-	-	-	-	-	-	-
Age years	r	0.115	-0.005	-0.031	-0.072	0.103	-0.131	-0.018	0.068
	p	-	-	-	-	-	-	-	-
MELD Score	r	0.090	**-0.223**	**-0.267**	**-0.314**	**-0.266**	**-0.378**	-0.182	**-0.229**
	p	0.235	**0.023**	**0.003**	**<0.001**	**0.003**	**<0.001**	-	**0.017**
ALT U/L	r	0.140	0.034	-0.089	-0.020	-0.007	-0.034	0.028	-0.166
	p	-	-	-	-	-	-	-	-
AST U/L	r	0.017	-0.134	**-0.246**	**-0.275**	**-0.231**	**-0.345**	-0.105	**-0.308**
	p	-	-	**0.008**	**0.002**	**0.016**	**<0.001**	-	**<0.001**
Bilirubin mg/dL	r	0.008	**-0.302**	**-0.348**	**-0.369**	**-0.385**	**-0.388**	**-0.328**	**-0.292**
	p	-	**<0.001**	**<0.001**	**<0.001**	**<0.001**	**<0.001**	**<0.001**	**<0.001**
Albumin g/L	r	-0.115	**0.265**	**0.252**	**0.359**	**0.316**	**0.441**	**0.227**	**0.218**
	p	-	**0.003**	**0.006**	**<0.001**	**<0.001**	**<0.001**	**0.020**	**0.030**
INR	r	0.194	-0.184	-0.191	**-0.282**	**-0.209**	**-0.360**	-0.133	-0.193
	p	-	-	-	**<0.001**	**0.042**	**<0.001**	-	-
Creatinine mg/dL	r	-0.051	-0.083	-0.134	-0.125	-0.051	-0.106	-0.092	0.004
	p	-	-	-	-	-	-	-	-
Leukocytes n/L	r	0.009	0.162	**0.283**	**0.270**	0.166	**0.294**	**0.252**	0.157
	p	-	-	**<0.001**	**<0.001**	0.028	**<0.001**	**0.001**	-
Platelets n/nL	r	-0.172	0.149	**0.331**	**0.303**	**0.222**	**0.408**	**0.241**	**0.235**
	p	-	-	**<0.001**	**<0.001**	**0.026**	**<0.001**	**0.011**	**0.014**
CRP mg/L	r	0.186	0.042	-0.013	-0.031	-0.056	-0.117	0.087	-0.064
	p	-	-	-	-	-	-	-	-
Ferritin ng/mL	r	0.030	0.025	0.064	0.042	-0.001	-0.053	0.070	-0.127
	p	-	-	-	-	-	-	-	-
HDL mg/dL	r	-0.027	0.018	-0.060	0.018	0.138	0.082	0.059	**0.217**
	p	-	-	-	-	-	-	-	**0.037**
LDL mg/dL	r	**0.327**	**0.529**	**0.615**	**0.666**	**0.596**	**0.636**	**0.548**	**0.329**
	p	**<0.001**	**<0.001**	**<0.001**	**<0.001**	**<0.001**	**<0.001**	**<0.001**	**<0.001**

Table S2. Spearman correlation coefficients and p-values for the correlation of ceramide species with BMI, age, MELD score, and routine laboratory parameters in the HCV patients without liver cirrhosis at therapy end. Significant correlations are in bold. – not significant (Alanine amino transferase (ALT), aspartate aminotransferase (AST), body mass index (BMI), C-reactive protein (CRP), high-density lipoprotein (HDL), international normalized ratio (INR), low-density lipoprotein (LDL), model of end stage liver disease (MELD).

Ceramide species d18:1;O2/ nmol/mL		16:0	18:0	20:0	22:0	23:0	24:0	24:1	26:0
BMI kg/m^2	r	0.062	0.054	0.036	0.118	0.095	0.056	-0.095	-0.095
	p	-	-	-	-	-	-	-	-
Age	r	0.153	0.172	0.167	0.174	**0.315**	0.098	0.144	0.217
	p	-	-	-	-	**<0.001**	-	-	-
MELD Score	r	-0.017	-0.024	0.007	0.004	-0.008	-0.036	0.012	-0.053
	p	-	-	-	-	-	-	-	-
ALT U/L	r	0.079	0.079	-0.083	0.030	0.029	0.006	0.033	-0.200
	p	-	-	-	-	-	-	-	-
AST U/L	r	0.051	0.040	-0.088	-0.040	-0.044	-0.097	-0.012	-0.200
	p	-	-	-	-	-	-	-	-
Bilirubin mg/dL	r	0.199	-0.228	**-0.239**	-0.189	**-0.272**	-0.183	**-0.294**	-0.197
	p	-	-	**0.040**	-	**0.011**	-	**0.004**	-
Albumin g/L	r	-0.003	0.128	0.026	0.098	0.137	0.186	0.147	0.100
	p	-	-	-	-	-	-	-	-
INR	r	0.098	0.000	0.117	0.048	0.083	0.006	0.082	0.002
	p	-	-	-	-	-	-	-	-
Creatinine mg/dL	r	0.034	0.077	-0.066	-0.094	-0.055	-0.112	-0.006	-0.006
	p	-	-	-	-	-	-	-	-
Leukocytes n/L	r	0.145	0.073	0.189	0.146	0.012	0.096	0.200	0.055
	p	-	-	-	-	-	-	-	-
Platelets n/nL	r	-0.130	-0.084	0.125	0.025	-0.035	0.104	0.066	0.098
	p	-	-	-	-	-	-	-	-
CRP mg/L	r	0.160	0.007	0.065	0.074	0.027	-0.013	0.099	-0.002
	p	-	-	-	-	-	-	-	-
Ferritin ng/mL	r	0.067	0.036	0.065	0.040	0.019	-0.054	0.070	-0.094
	p	-	-	-	-	-	-	-	-
HDL mg/dL	r	-0.107	-0.012	-0.073	0.003	0.114	0.091	0.044	0.220
	p	-	-	-	-	-	-	-	-
LDL mg/dL	r	**0.502**	**0.506**	**0.555**	**0.574**	**0.506**	**0.512**	**0.513**	0.210
	p	**<0.001**	**<0.001**	**<0.001**	**<0.001**	**<0.001**	**<0.001**	**<0.001**	-

Table S3. Spearman correlation coefficients and p-values for the correlation of ceramide species with BMI, age, MELD score, and routine laboratory parameters in the HCV patients with liver cirrhosis at therapy end. Significant correlations are in bold. – not significant (Alanine amino transferase (ALT), aspartate aminotransferase (AST), body mass index (BMI), C-reactive protein (CRP), high-density lipoprotein (HDL), international normalized ratio (INR), low-density lipoprotein (LDL), model of end stage liver disease (MELD).

Ceramide species d18:1;O2/ nmol/mL		16:0	18:0	20:0	22:0	23:0	24:0	24:1	26:0
BMI kg/m^2	r	-0.071	0.007	0.102	0.060	0.072	0.154	0.157	0.043
	p	-	-	-	-	-	-	-	-
Age years	r	-0.337	-0.162	-0.075	-0.233	-0.137	-0.126	-0.233	0.014
	p	-	-	-	-	-	-	-	-
MELD Score	r	-0.002	-0.387	-0.401	-0.408	**-0.521**	**-0.510**	-0.388	-0.208
	p	-	-	-	-	**0.004**	**0.005**	-	-
ALT U/L	r	0.091	-0.115	-0.125	-0.183	-0.108	-0.187	0.038	-0.085
	p	-	-	-	-	-	-	-	-
AST U/L	r	-0.134	-0.229	-0.078	-0.235	-0.313	-0.339	0.097	-0.296
	p	-	-	-	-	-	-	-	-
Bilirubin mg/dL	r	0.071	-0.261	-0.269	-0.351	**-0.426**	-0.387	-0.288	-0.232
	p	-	-	-	-	**0.043**	-	-	-
Albumin g/L	r	-0.182	0.311	0.284	0.323	0.355	0.396	0.076	0.104
	p	-	-	-	-	-	-	-	-
INR	r	0.128	-0.224	**-0.438**	**-0.468**	**-0.612**	**-0.651**	**-0.434**	-0.339
	p	-	-	**0.033**	**0.016**	**<0.001**	**<0.001**	**0.036**	-
Creatinine mg/dL	r	-0.373	-0.412	-0.198	-0.071	0.089	0.127	-0.215	0.186
	p	-	-	-	-	-	-	-	-
Leukocytes n/L	r	-0.111	-0.011	0.126	0.159	0.265	0.311	0.083	0.120
	p	-	-	-	-	-	-	-	-
Platelets n/nL	r	-0.080	0.232	0.264	0.328	0.381	**0.452**	0.257	0.147
	p	-	-	-	-	-	**0.027**	-	-
CRP mg/L	r	0.197	0.276	0.019	-0.017	-0.117	-0.141	0.193	-0.107
	p	-	-	-	-	-	-	-	-
Ferritin ng/mL	r	-0.063	0.122	0.192	0.154	0.064	0.085	0.138	-0.200
	p	-	-	-	-	-	-	-	-
HDL mg/dL	r	0.119	0.210	0.156	0.371	0.404	0.330	0.266	0.308
	p	-	-	-	-	-	-	-	-
LDL mg/dL	r	0.290	0.380	**0.497**	**0.646**	**0.655**	**0.652**	**0.496**	0.414
	p	-	-	**0.010**	**<0.001**	**<0.001**	**<0.001**	**0.011**	-

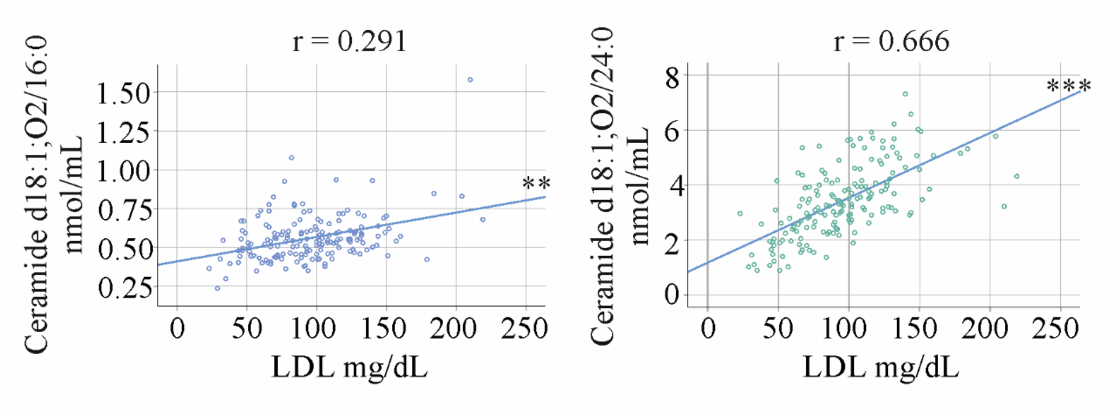

Figure S1. Correlation of ceramides and LDL in the whole cohort before therapy. (**a**) Correlation of ceramide d18:1;O2/16:0 with LDL; (**b**) Correlation of ceramide d18:1;O2/24:0 with LDL. ** $p < 0.01$; *** $p < 0.001$.

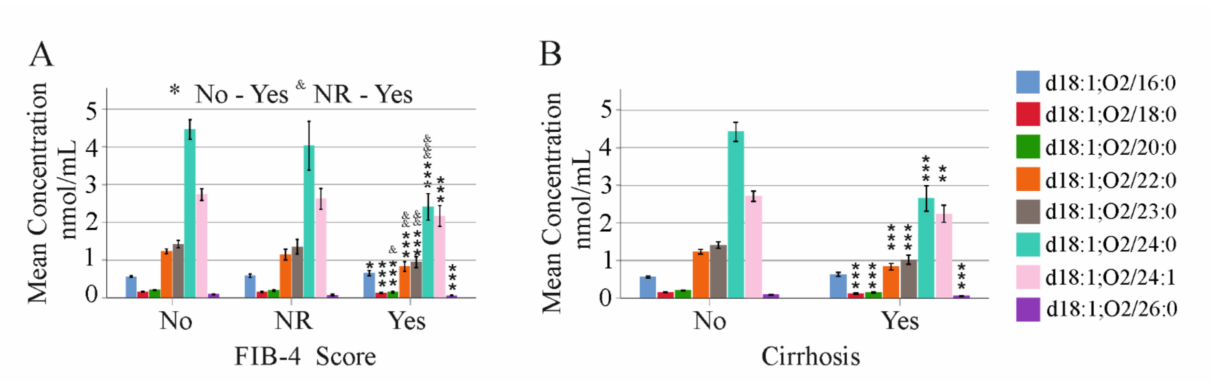

Figure S2. Ceramide species at therapy end in relation to the FIB-4 score and cirrhosis diagnosed by ultrasound. (**a**) Mean concentration ± 95% confidence interval of ceramide levels in patients stratified for fibrosis by the FIB-4 score, (no fibrosis = No, 109 patients; not reliable values = NR, 33 patients; fibrosis = Yes, 31 patients); (**b**) Mean concentration ± 95% confidence interval of ceramide species in patients without (No, 136 patients) and with (Yes, 41 patients) liver cirrhosis diagnosed by ultrasound. * $p < 0.05$, ** $p < 0.01$, *** $p < 0.001$, & $p < 0.05$, && $p < 0.01$, &&& $p < 0.001$.
